# A Self-Supervised Few-Shot Semantic Segmentation Method Based on Multi-Task Learning and Dense Attention Computation

**DOI:** 10.3390/s24154975

**Published:** 2024-07-31

**Authors:** Kai Yi, Weihang Wang, Yi Zhang

**Affiliations:** 1 Intelligent Policing Key Laboratory of Sichuan Province, Luzhou 646099, China; yikai@scpolicec.edu.cn; 2 College of Computer Science, Sichuan University, Chengdu 610042, China; wei_sailing@163.com

**Keywords:** scene understanding, self-supervised learning, few-shot semantic segmentation, self-supervised learning, multi-task learning, Swin Transformer

## Abstract

Nowadays, autonomous driving technology has become widely prevalent. The intelligent vehicles have been equipped with various sensors (e.g., vision sensors, LiDAR, depth cameras etc.). Among them, the vision systems with tailored semantic segmentation and perception algorithms play critical roles in scene understanding. However, the traditional supervised semantic segmentation needs a large number of pixel-level manual annotations to complete model training. Although few-shot methods reduce the annotation work to some extent, they are still labor intensive. In this paper, a self-supervised few-shot semantic segmentation method based on Multi-task Learning and Dense Attention Computation (dubbed MLDAC) is proposed. The salient part of an image is split into two parts; one of them serves as the support mask for few-shot segmentation, while cross-entropy losses are calculated between the other part and the entire region with the predicted results separately as multi-task learning so as to improve the model’s generalization ability. Swin Transformer is used as our backbone to extract feature maps at different scales. These feature maps are then input to multiple levels of dense attention computation blocks to enhance pixel-level correspondence. The final prediction results are obtained through inter-scale mixing and feature skip connection. The experimental results indicate that MLDAC obtains 55.1% and 26.8% one-shot mIoU self-supervised few-shot segmentation on the PASCAL-$5^{i}$ and COCO-$20^{i}$ datasets, respectively. In addition, it achieves 78.1% on the FSS-1000 few-shot dataset, proving its efficacy.

## 1. Introduction

With the rapid improvement of computing power (supported by advanced hardware platforms) and the rise of deep learning algorithms, various scene awareness schemes have been integrated into smart cars. For instance, Light Detection and Ranging (LiDAR), Radio Detection and Ranging (RADAR) are 2 important auxiliary equipment for scene understanding under poor lighting conditions via radio waves and laser pulses, respectively [1]. They are highly valued for precise distance measurement (for scene reconstruction and mapping), but behave poor in visual recognition aspect. Ultrasonic sensors are specially designed for parking assistance, but they are limited to short distance warning. By contrast, vision sensors with the state-of-the-art recognition and perception algorithms greatly improve the scene awareness ability of the intelligent vehicles, which also have lower prices than the abovementioned instruments. The commonly involved algorithms in scene understanding include object detection, semantic segmentation and depth recovery [2] etc.

Traditional deep learning approaches often require a massive number of labeled samples. Computer vision tasks like image segmentation typically need numerous high-quality pixel-level annotations to guide model training [3,4]. However, acquiring such annotated data is expensive, time-consuming, or even infeasible. Few-shot learning leverages prior knowledge to generalize to new tasks with only a few supervised samples. Therefore, to reduce the labeling costs of image segmentation under scenarios with limited or scarce samples, many studies have incorporated few-shot learning into the image semantic segmentation field.

Few-Shot Semantic Segmentation (FSS) requires less annotation data to complete pixel-level semantic segmentation tasks, improving its generalization ability. Normally, a limited number of images is annotated for one category, which indicates that the model must learn intra-class features and migrate them to unseen classes. Currently, mainstream FSS includes metric learning-based methods, parameter prediction-based methods, fine tuning-based methods, and memory-based methods. Among them, metric learning-based methods [5,6,7,8,9] play a dominant role. The distances between feature vectors supporting images and querying images in high-dimensional space are used [5] to calculate the similarity between them so as to predict the category probability of each pixel in the image.

In this method, the amount of annotation of images without visible categories is greatly reduced, but the annotation requirement of visible categories is still indispensable in the training process. Therefore, it is still challenging to further reduce annotation requirements using few-shot semantic segmentation. A two-stage unsupervised image segmentation method was proposed by the authors of [10], who used the K-means clustering method to cluster pixels in images into semantic groups to obtain significant regions with continuous semantic information. A self-supervised FSS method based on unsupervised significance for prototype learning was devised in [11] to generate training pairs from a single training image to capture the similarity between the queried image and a specific region supporting the image. The feature vector of the Laplacian matrix derived from the feature affinity matrix of a self-supervised network was utilized in [12] to eliminate the need for extensive annotation, effectively capturing the global representation of the object of interest in the supporting image. As mentioned earlier, traditional methods rely heavily on manual annotation. Although the above methods alleviate such problems to some extent under a self-supervised learning framework, most of them do not fully utilize the different scale features, leading to poor segmentation performance.

To solve the abovementioned problem, a self-supervised few-shot segmentation method based on saliency segmentation is proposed in this paper. Our method is built under a multi-task learning framework. Each saliency mask is divided into two parts, one of which is used as a support mask in a random way, while the other part is used as a query mask to participate in the model training of few-shot meta learning. To further enhance the meta learning effect, multiple learning tasks are proposed after saliency segmentation to jointly enhance the few-shot segmentation performance. At the same time, in order to enhance the robustness of the model, noise addition and image enhancement are applied to process the input image so as to better simulate FSS tasks. To fully utilize the multi-scale features, a dense attention calculation mechanism is developed, which transforms the multi-scale feature map into a multi-scale dense attention block to yield the final prediction result via inter-scale mixing. Finally, the self-supervised few-shot semantic segmentation method is formed, which is based on a multi-task learning scheme and dense attention calculation.

The main contributions of this paper are summarized as follows:

1. A self-supervised few-shot segmentation method is proposed based on a multi-task learning paradigm. The unsupervised salient part of the image is split into two parts; one of them is used as a support image mask for few-shot segmentation, and the other part and the entire image are used to calculate the cross-entropy with the prediction results to realize multi-task learning so as to improve the generalization ability;
2. An efficient few-shot segmentation network based on dense attention computation is proposed. Multi-scale feature extraction is carried out using Swin Transformer so as to make full use of the multi-scale pixel-level correlation.

Experimental results obtained on three mainstream datasets show that our method surpasses other popular methods in segmentation accuracy, demonstrating its efficacy.

## 2. Related Works

### 2.1. Few-Shot Semantic Segmentation with Fully Supervised Learning

A two-branch network with a prediction-based method was proposed in [13], which consists of a conditional branch and a segmentation branch. It aims to solve a one-way one-shot image segmentation problem by using parameter prediction-based methods to modify the classifier weights for cross–class adaptation. Instead of relying only on support samples, query images were also used to generate classifier weights [14]. Instead of directly replacing the classifier parameters, the classifier weights were dynamically added in [15], enabling the model to master both base and unseen categories.

Metric learning-based methods are the most commonly used techniques for FSS. Among them, the methods based on prototype networks are particularly prevalent. In traditional learning-based methods [8,16], the learned prototype of a class is an approximate estimate of the optimal prototype. Recent few-shot methods aim to obtain specific prototypes of objects so as to provide relevant information. Such methods provide higher similarity scores for query characteristics belonging to the same semantic classes as the object instead of approximating the best prototype [5,6,17,18]. When applying the masked average pooling operation to generate a holistic descriptor for each semantic category in these methods, some problems arise, such as the prototype learner failing to output a robust class representation, making it difficult to capture rich and fine-grained semantic information only using a global feature vector due to the visual differences between support and query images. To address these issues, some subsequent approaches have sought to generate multiple prototypes for each semantic category [9,19], and others perform intensive matching between support and query images [20,21,22]. The fine tuning-based approaches aim to use an optimization algorithm to improve the parameters of the pre-trained segmentation network to learn unseen categories [23,24,25]. In memory-based FSS, semantic information is retained to assist in segmentation of query samples and obtain more cross-resolution information and precise segmentation results [26,27].

### 2.2. Self-Supervised Learning for Image Semantic Segmentation

SSL bridges supervised learning and unsupervised learning. It still requires large-scale labeled data to obtain better performance in terms of visual features. To avoid high-cost annotation, methods are proposed to learn general image and video features from unlabeled data without additional supervision. Self-supervised learning aims to obtain universal image or video features by learning large-scale unlabeled data without any manual annotation. In image segmentation, self-supervised segmentation refers to the automatic generation of segmentation labels for images without the need for manual annotation in order to achieve learning of image segmentation tasks.

The self-supervised semantic segmentation model predicts a set of labels (i.e., masks with deterministic meanings) based on the input data. Previous methods [28,29] were based on offline pre-calculated labels, followed by model updating. As a more lightweight method, self-training on pseudo-labels is an important way to seek high-quality supervision through high-confidence class prediction. Wen et al. [30] defined each class of objects as a learnable class vector and calculated the similarity between the class vector and each position in the image feature map. They aggregated features of the same class in the image, then constructed positive and negative sample pairs of the aggregated class features for comparative training and learning. Araslanov et al. [31] applied standard data augmentation techniques such as noise, flipping, and scaling to self-supervised segmentation, ensuring consistent semantic prediction results across different image transformations.

### 2.3. FSS Vision Transformers

The use of transformers was originally proposed for natural language processing tasks due to their excellent long-range dependency modeling ability. They were later migrated to the computer vision domain [32,33].

The combination of a vision transformer and FSS is a recently emerging topic. Lu et al. [34] designed a Classifier Weight Transformer (CWT) to dynamically adjust the weight of the classifier for each query image to make better use of the support set (a limited collection of images with corresponding annotated masks to furnish the model with exemplars of the target classes) in the query image. However, it still follows the prototype pipeline and, therefore, cannot fully exploit the supporting information at a fine-grained level. A novel Cyclic Consistent Transformer (CyCTR) module was developed in [35] that aggregates pixel-level support features into query features, focusing on providing each query pixel with relevant information from the support image to facilitate the classification of query pixels. DCAMA [36] follows the design of CyCTR, introducing full exploitation of the support image by pairing queries and supporting multi-level pixel-level correlations between features.

## 3. Method

In this section, we introduce our proposed MLDAC in detail. First, the task of self-supervised few-shot segmentation is clarified; then, our multi-task framework is introduced in Section 3.2. The core modules in MLDAC are described in Section 3.3.

### 3.1. Problem Definition

**Fully supervised few-shot semantic segmentation.** Traditional FSS is always based on fully supervised learning. Specifically, given the same class of images and corresponding mask conditions in the training set ($D_{train}$), the model aims to find the designated area related to the mask in another image based on the images and corresponding masks in the given test set ($D_{test}$) so as to accomplish the few-shot segmentation task. This is the meta-learning paradigm called episodic training. In real applications, both $D_{train}$ and $D_{test}$ consist of different classes of objects, and image pairs with the same category are selected to realize the meta-learning paradigm. $D_{train}$ over class $C_{train}$ has a completed annotation mask for every image. The classes ($C_{test}$) of $D_{test}$ have no shared classes, as is the case for $C_{train}$ (i.e., $C_{train}$∩$C_{test}$ = Φ). In episodic training, each image pair contains a duplicate image, mask, and class information, where the class information is the same, i.e., for $\left(x_{1},m_{1},y_{1}\right)$ and $\left(x_{2},m_{2},y_{2}\right)$, $\left(y_{1}=y_{2}\right)$, where $x_{1}$ and $x_{2}$ are the images, $m_{1}$ and $m_{2}$ are the ground-truth binary masks, and $y_{1}$ and $y_{2}$ are the class labels corresponding to the mask.

**Self-supervised few-shot semantic segmentation (SFSS).** For the self-supervised few-shot semantic segmentation problem, $D_{train}$ consists of images without masks or labels so that the training process cannot be implemented. To solve this problem, a new SFSS method based on multi-task learning to build a self-supervised experimental process is proposed. After training, the same evaluation protocol as the standard FSS can be used to evaluate the learned meta-models for a multitude of segmentation tasks with few images.

### 3.2. Framework

**Figure 1:**
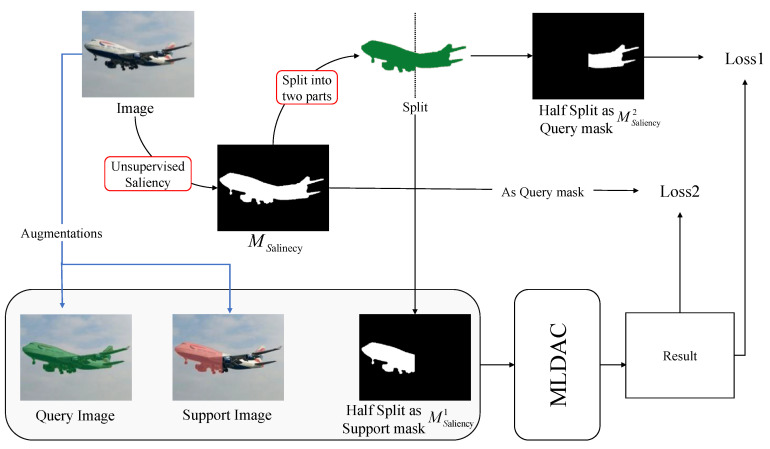
The overall structure of the proposed self-supervised network. The unsupervised saliency mask is segmented; one part is used for masking and support, and the other part and the entire unsupervised salient area are used to calculate the loss function so as to guide model training.

To realize SFSS, a complete episodic training framework is constructed in this paper. The architecture of our proposed MLDAC based on multi-task learning consists of three inputs (query image, support image, and support mask) and one output (segmentation result), as shown in Figure 1. The input is a single image without any annotation or class label ($I_{image}$∈$D_{train}$). Self-supervised learning usually uses data attributes to set unsupervised tasks instead of manual annotation. Therefore, unsupervised saliency prediction was utilized to obtain the saliency region ($M_{saliency}$), which depicts the arbitrary object in the image with continuous and accurate edge information. Next, $M_{saliency}$ is divided into 2 parts, namely $M_{saliency}^{1}$ and $M_{saliency}^{2}$, the former of which is used as the support mask that is input to MLDAC, while $M_{saliency}^{2}$ and $M_{saliency}$ are used to calculate the loss as follows:

(1) $$loss1=CrossEntropyLoss\left(M_{saliency}^{1},result\right);ignore\;\_index=M_{saliency}$$

(2) $$loss2=CrossEntropyLoss\left(M_{saliency},result\right);ignore\;\_index=\Phi ;$$

then,

(3) $$result=MLDAC(I_{Query},I_{Support},M_{saliency}^{2})$$

Equations (1) and (2) are both cross-entropy loss functions with slightly different implementation details. For Loss 1, $M_{saliency}^{1}$ do not participate in the calculation of the loss function so that the model focuses on learning the query region, weakening the impact of the support region. Loss1 and Loss2 guide model training in an alternate way, with probabilities set to a and 1-a, respectively.

Meanwhile, since $I_{Query}$ and $I_{Support}$ come from the same source, to highlight the difference between them, we employ data augmentation techniques, including jittering, horizontal flip-flop, rotation, and random cropping. Gaussian noise is also added before image enhancement (i.e., the color of the selected query region is perturbed slightly to augment the diversity of the training data). The pseudo code of our proposed self-supervised few-shot semantic segmentation framework is expressed in Algorithm 1 as follows:
**Algorithm 1 **FSS self-supervised framework based on multi-task learning1:multi-task_split(Image):2:Saliency= Unsupervised_Saliency _ Detection(Image)3:Saliency1,Saliency2=Split(Saliency)4:**if **random.random()<a** then**5:    target=Saliency6:    loss=nn.CrossEntropyLoss()7:**else **{}8:    target=Saliency29:    loss=nn.CrossEntropyLoss(gnore_index=Saliency1)10:**end if**11:q_image,target=Augmentations(Image,target)12:s_image,s_mask=Augmentations(Image+b,Saliency1)13:result=MLDAC(q_image,s_image,s_mask)14:loss=loss(result,target)

### 3.3. MLDAC Network Architecture

**Figure 2:**
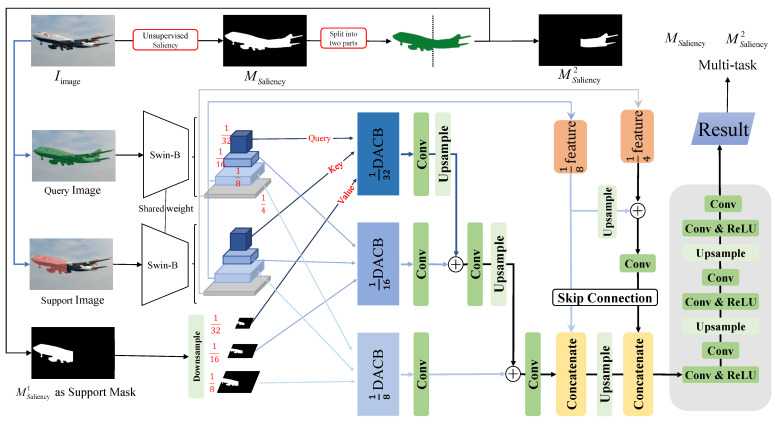
The architecture of our network with the proposed self-supervised meta-learning approach.

As shown in Figure 2, our proposed MLDAC consists of the following 3 parts:

In the first part, a pre-trained feature extractor is applied to process both the query and the support images to obtain multi-scale query and support features and support image masks of the corresponding size;

The second part inputs the query features, support features, and support masks at each scale into a multi-layer Dense Attention Computation Block (DACB) of the same scales as Q, K, and V. DACB performs a multi-scale query, support features, and support mask attention calculations;

The third part involves the aggregation of the outputs from the previous stage and the multi-scale features. This process produces the final prediction masks using a tailored mixer.

#### 3.3.1. Feature Extraction and Masking

The first stage involves the extraction of different levels of semantic features. Here, Swin Transformer (Swin-B) is employed as the feature extractor, which captures both local fine-grained features and contextual semantic information. Through the bottom-up pathway, features at multiple scales are computed, enabling multi-scale feature learning. Following [7], after capturing image features of different sizes, the image mask is scaled to different support mask sizes via linear interpolation, allowing for cross-feature attention in different layers. Compared with existing FSS models, the Swin Transformer model (Swin-B) is adapted to extract features and was pre-trained on ImageNet-1K and liu2021swin.

#### 3.3.2. Dense Attention Computation Block(DACB)

Our proposed DACB aggregates multi-scale features to produce semantic information. The initial stage involves the transformer architecture (i.e., scaled dot-product attention). The corresponding calculation is written as follows:

(4) $$Attn\left(Q^{\prime },K^{\prime },V\right)=softmax\left(\frac{Q^{\prime }{K^{\prime }}^{T}}{\sqrt{d}}\right)V,$$

where Q,K, and *V* are the sets of query, key, and value vectors, respectively; d represents the dimension of the query and key vectors; and $Q^{\prime },K^{\prime }$ indicates that the location code has been added to Q,K with absolute learnable position encoding.

**Figure 3:**
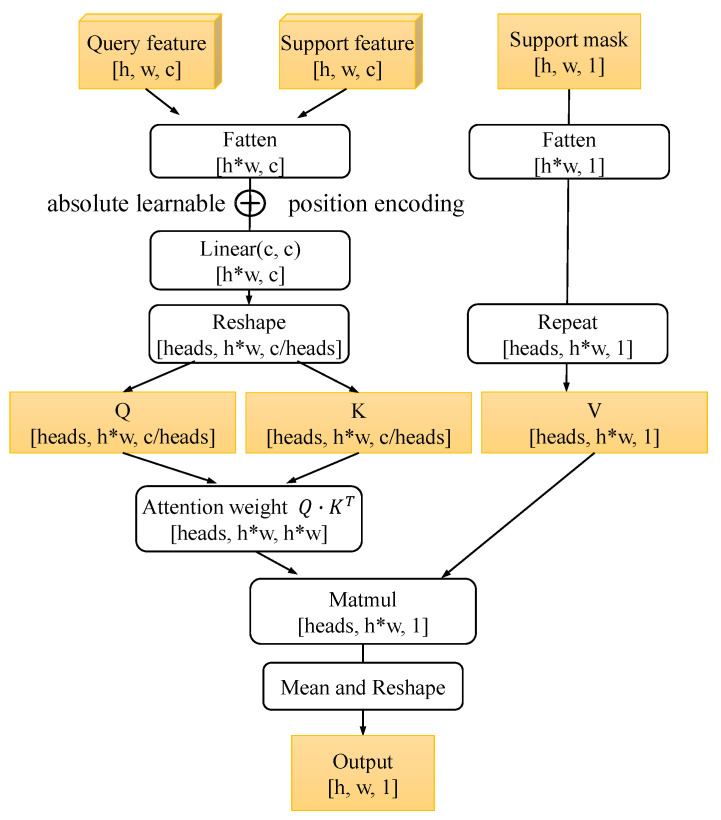
Illustration of the proposed DACB.

In this paper, the query and support feature maps are denoted as $F^{q}$, $F^{s}$∈$R^{h\times w\times c}$, where *h*, *w*, and *c* represent the height, width, and number of channels of the feature maps, respectively. As shown in Figure 3, the support feature (Fq) and query feature (Fs) are flattened first, and each pixel value is regarded as a token. Then, after adding learnable linear position coding, the $Q^{\prime }$, $K^{\prime }$ matrix is generated from the flattened $F^{q}$ and $F^{s}$, and the multi-head attention mechanism is implemented as follows:

(5) $$MHA\left(Q^{\prime },K^{\prime },V\right)=\left[head_{1},head_{2},\cdots ,head_{n}\right],$$

where $head_{m}=Atten\left(Q_{m}^{\prime },K_{m}^{\prime },V_{m}\right)$, and the inputs $\left[Q_{m}^{\prime },K_{m}^{\prime },V_{m}\right]$ are the $m^{th}$ group from $\left[Q_{m}^{\prime },K_{m}^{\prime },V_{m}\right]$ with dimension d/h. For the support mask, it is only necessary to flatten it to participate in the calculation of dense attention. Finally, the output of the multiple attention heads of each token is averaged, and the averaged output is reset to a two-dimensional tensor expressed as $\hat{R}$ with dimensions of h×w×c, which is the final dense attention computation output.

#### 3.3.3. Inter-Scale Mixing and Up-Sampling Module

After cross-feature dense attention computation at different scales of features from multiple layers, it is necessary to mix attention results from these different scales to obtain the final prediction. Our inter-scale mixing and up-sampling module has 2 parts; one stitches the different layers directly after cross-feature dense attention computation, and the other one improves the recognition of image features using skip linking. In this step, the size of each layer is adjusted via continuous up-sampling operations.

First, the dense attention computation scale of $\frac{1}{8},\frac{1}{16},\frac{1}{32}$ is specifically used to obtain the attention-weighted result, and $R_{1/8},R_{1/16},R_{1/32}$. $R_{1/i}$ are subsequently processed through several convolution blocks that are finally merged into the resultant block after suitable up-sampling operations. The outputs of 1/32 and 1/16 are connected, resized, and concatenated to yield the outputs of the 1/8 scale as follows:

(6) $$R_{1/32+1/16}^{\prime }=\left(↑k*R_{1/32}^{\prime }\right)⊕k*R_{1/16}^{\prime }$$

(7) $$R^{\prime }=\left(↑k*R_{1/32+1/16}^{\prime }\right)⊕k*R_{1/8}^{\prime }$$

where ↑ is the up-sampling operation and ⊕ stands for the connection operation. $R^{\prime }$ is then processed by a skip connection and decoding operation to obtain the final predicted mask. Then, the last layer of features extracted by the feature extractor with 1/4 and 1/8 scales are concatenated as follows:

(8) $$R^{\prime \prime }=R^{\prime }⊕F_{1/8}^{q}⊕F_{1/8}^{s}$$

(9) $$F_{1/8+1/4}=↑F_{1/8}^{q}⊕↑F_{1/8}^{s}⊕F_{1/4}^{q}⊕F_{1/4}^{s}$$

(10) $$R^{\prime \prime \prime }=↑R^{\prime \prime }⊕\left(k*F_{1/8+1/4}\right)$$

Finally, the result is obtained using a decoder ($f\left(X\right)$) to produce the final mask prediction as follows:

(11) $$M_{result}=f\left(R^{\prime \prime \prime }\right)$$

The decoder is composed of several convolutional modules and ReLU blocks that operate alternately, along with up-sampling operations to attain the final segmentation resolution. The decoder blocks gradually reduce the dimensions of the output channels to 2 (1 for foreground and the other for background) in one-way segmentation. Two interleaved up-sampling operations are used to restore the output size to match that of the input images.

## 4. Experiments and Results

**Datasets.** To validate the effectiveness of our proposed method, extensive experiments were conducted on the PASCAL-$5^{i}$, COCO-$20^{i}$ and FSS-1000 datasets.

PASCAL-$5^{i}$ is built upon PASCAL VOC [37]. It has 20 categories that are further divided into 4 folds, namely $5^{0}$, $5^{1}$, $5^{2}$, and $5^{3}$. Each fold has different kinds of categories. For instance, $5^{0}$ includes planes, bikes, birds, etc., while $5^{1}$ includes buses, cars, chairs, etc. During the training for each fold, the other three folds are used as the training dataset. We need only image data for our unsupervised training, without the tasks or class information associated with the images. Hence, we use images from all the folds to support our training and use the unsupervised saliency map of all folds and the folded images to assess the average concurrency ratios of each fold and preserve the best outcomes.

Similar to PASCAL-$5^{i}$, COCO-$20^{i}$ is derived from MS COCO [38], which consists of more than 120,000 images from 80 categories. It is split into four folds denoted by $20^{0}$, $20^{1}$, $20^{2}$, and $20^{3}$, each of which contains 20 categories.

The FSS-1000 dataset [39] is set up with well-established categories; we use only images from pre-trained categories as support and do not use images from the target categories as part of the training set. For all datasets, the mean intersection over union (mIoU) is used, and one-shot segmentation results are reported and compared.

### 4.1. Implementation Details

All experiments were performed using PyTorch framework. The pre-trained Swin-B-based model is used as the backbone feature extractor, (which is trained on ImageNet-1K [33]). Both support and query images have input sizes of 384×384 pixels. For optimization, rgw Adam optimizer was applied with a learning rate of $10^{-4}$, a weight decay of $10^{-5}$, and pixel cross-entropy loss. Each model was trained on two 3090 GPUs for 100 epochs using the PASCAL dataset and 30 epochs using the COCO dataset, with a batch size of 16.

### 4.2. Comparison with Other Popular Methods

Comparisons are made in Table 1 and Table 2 between our method and other state-of-the-art supervised few-shot segmentation approaches and self-supervised semantic segmentation approaches. Here, avg represents the mean intersection over union, $5^{i}$ represents the average category segmentation accuracy of all categories in the i-th fold, and FSS-1000 represents the segmentation accuracy on the FSS-1000 dataset. The supervised models utilized the ground-truth segmentation mask during usual fold-based training, whereas the unsupervised models were trained on a training set without the ground truth. As shown in Table 1, we achieved the best results among all the self-supervised methods and even surpassed two of the fully supervised methods. Similarly for COCO and FSS-1000, we also achieved the best overall results among all the self-supervised methods, exceeding two of the fully supervised methods (on COCO).

Above all, the framework that we propose is highly effective. When comparing results on the PASCAL dataset, we use the results reported in MASKSPLIT, where the Saliency* and MaskContrast* methods are from [10], optimized to obtain unsupervised approaches that represent the framework. In order to make a comprehensive comparison with the existing supervised few-shot method, the source code provided by MIANet [42] is used to re-conduct the experiment under the self-supervised settings, obtaining a result of 53.8%. In comparison to supervised approaches, self-supervised approaches perform poorly because they do not learn intra-class information. Despite this, our approach performed exceptionally well, achieving a score of 55.1% on PASCAL. This score is two points higher than the initial slicing approach, MASKSPLIT, which is very competitive.

Table 2 shows a comparison between our method and other popular self-supervised and fully-supervised few-shot segmentation methods on COCO and FSS-1000. As shown by the results, the performance of our method was greatly enhanced compared to current self-supervised few-shot segmentation methods, with an increase from 23.3 to 26.8 on the COCO dataset and to 78.1 on the FSS-1000 dataset, which is a significant improvement. We attribute the superb results on the FSS-1000 dataset to the unsupervised saliency regions being more prominent and free of noise and to the relatively high within-class image similarity. It is worth noting that MaskSplit surpasses our method on $5^{3}$ in both Table 1 and Table 2. The reason is that MaskSplit masks out all the background regions of the supported image (i.e., masked pooling) during the self-supervised training process. This strategy is effective when facing a complex background. However, we realize better overall results by using dense attention computation to acquire both the background and foreground information.

To further demonstrate the advantages of our model for cross-domain few-shot segmentation tasks, an additional experiment was conducted on the ISIC2018 dataset [44], which contains skin lesion images and is mainly used for medical image analysis and model training. It comprises thousands of high-resolution images of skin lesions, including benign lesions such as moles and pigmented nevi, as well as malignant lesions such as melanoma and basal cell carcinoma. The major advantage is that the images have already been annotated by professionals.

Comparative results on ISIC are shown in Table 3. PATNet [45] proposes a few-shot segmentation network based on pyramid anchoring transformation, which converts domain-specific features into domain-independent features for downstream segmentation modules to quickly adapt to unknown domains. PMNet [46] proposes a pixel matching network that extracts domain-independent pixel-level dense matches and captures pixel–pixel patch relationships in each supporting query pair using bidirectional 3D convolution. Compared with PATNet [45] and PMNet [46], we achieved much higher accuracy.

### 4.3. Analysis of the Computational Complexity

In this section, we analyze the computational complexity of MLDAC in terms of model parameters (Params), floating-point operations (FLOPs), and inference time. Among them, Params indicates the number of parameters that the model has (i.e., model size), and FLOPs indicates the computation cost during inference. The inference time is the time that the model spends to produce the segmentation results. The experiment was conducted on 2 RTX 3090 GPUs, and we adopted Swin-B as our backbone. A comparison is shown in Table 4 below. Compared with HSNet [7], although our method has higher computational costs, it requires fewer iterations (i.e., much shorter inference time).

### 4.4. Visualization Results

**Figure 4:**
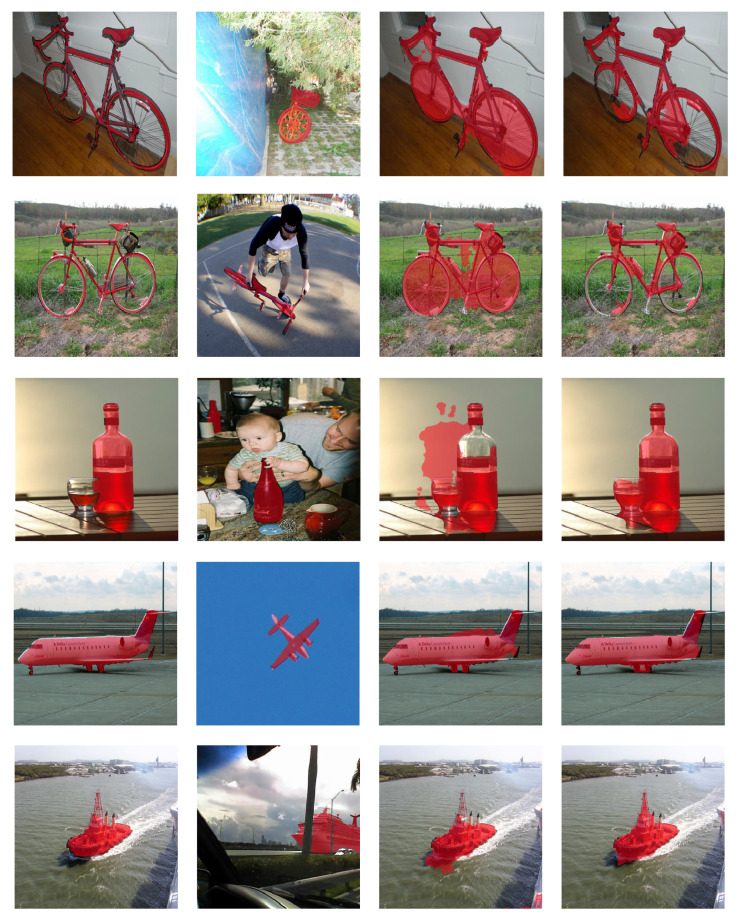
Comparison of visualization results on PASCAL-$5^{i}$. Columns correspond to the query image with mask, support image with mask, MaskSplit results, and our results.

In this section, a comparison of visualization results is shown to demonstrate the segmentation results obtained by different methods. As shown in The first column in Figure 4 shows the ground-truth segmentation results; the second column shows the supporting image and corresponding masks; and the third and the fourth columns show the segmentation results obtained by MaskSplit and our method, respectively. In comparison, our proposed method clearly delineates the boundary of each object better than MaskSplit. For complex backgrounds, MLDAC can better distinguish the foreground and the background based on the supporting images.

### 4.5. Ablation Study

A comprehensive ablation study was conducted on PASCAL to validate the effectiveness of our proposed method.

#### 4.5.1. Multi-Task Learning Parameter Settings

**Figure 5:**
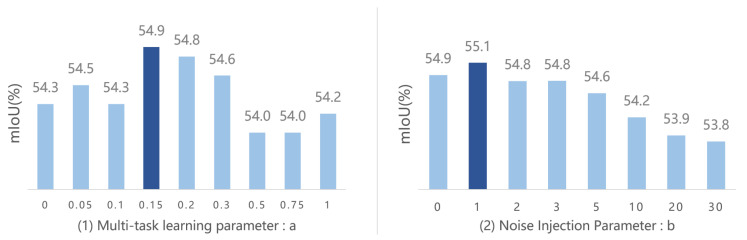
Ablation experiments on the value of parameters a and b.

In this section, the proposed multi-task learning parameter (a) and noise injection parameter (b) in MLDAC are described. Here, *a* represents the probability of selecting task 1 or task 2 during training, which balances the proportion of the two few-shot segmentation target loss functions. When it is offset to 0 or 1, the network degenerates into an ordinary single-target structure. The experimental results obtained using different values of a are shown in Figure 5(1). It can be seen that when a = 0.15, the model achieves the optimal result; therefore, we set a = 0.15 for all other experiments. Similarly, as shown in Figure 5(2), the best result is reached when b=1. Here, *b* represents the mean of Gaussian noise mixed into the image with additive noise.

#### 4.5.2. The Architecture of MLDAC

As shown in Table 5, the combination of different schemes was validated to search for the optimal settings of MLDAC. As shown in Table 5, our proposed learnable linear positional encoding and skip connection are, indeed, effective. The former enhances the connections between different features, and the latter strengthens the semantic information, making it easier to obtain the correlated region between the supporting and query images. Meanwhile, the 1/4 and 1/8 features accomplish the segmentation task in a more effective way.

It can be seen from the data in Table 5 that multi-scale DACBcan better capture semantic information of different scales and obtain better segmentation effects than the compared models. Removing 18 scale-intensive attention computing blocks reduces the model performance by 0.6%, and consecutive removal of $\frac{1}{8}$ and $\frac{1}{16}$ level dense attention computation blocks reduces the model’s performance.

#### 4.5.3. Configuration of Learnable Absolute PE and Dense Skip Connections

Ablation studies were performed on the absolute learnable positional encodings and skip connections, and the results are shown in Table 6. The absolute learnable position encoding we use is added only at the $\frac{1}{16}$ and $\frac{1}{32}$ levels of dense attention computation. The $\frac{1}{8}$ level uses the encodings fixed by sine and cosine functions with different frequencies to save training costs. Our skip connections of the $\frac{1}{4}$ scale refer to the up-sampling of features from the previous layer when using features from the later layer and performing the original skip-linking operation by using a conv module to resize the spliced features back to the original size. Ablation experiments show that absolute learnable position encoding facilitates the experiments. Using the dense skip connection operation only on $\frac{1}{8}$ and $\frac{1}{4}$ can improve the final segmentation by identifying the features in the intermediate layer more efficiently. The combination of $\frac{1}{4}$ scale features and $\frac{1}{8}$ scale features with conv blocks before skip connections can complete segmentation tasks more efficiently.

## 5. Conclusions

Self-supervised methods have begun to prevail in multiple computer vision tasks, including semantic segmentation. In this paper, a self-supervised few-shot segmentation method is proposed based on multi-task learning and dense attention computation. Our method utilizes unsupervised saliency regions for self-supervised learning for few-shot segmentation (FSS), which avoids the need for extensive manual annotation. The unsupervised saliency regions provide continuous semantic information to improve the training of self-supervised FSS. The self-supervised FSS method based on multi-task learning is proposed to solve the lack of category information, which divides the salient regions into query regions and support regions. The introduction of an attention mechanism improves the segmentation accuracy of the model. Extensive experiments were conducted on COCO-$20^{i}$ and PASCAL-$5^{i}$, on which our model achieved 55.1% and 26.8% one-shot mIoU, respectively. In addition, it realized 78.1% on FSS-1000.

Despite the appealing results we achieved, the proposed self-supervised FSS method based on saliency segmentation still cannot effectively provide continuous salient regions for objects of the same category. In the future, we plan to introduce an image generation scheme to construct a meta-learning paradigm for FSS so as to achieve higher segmentation accuracies.

## Figures and Tables

**Table 1:** Comparison of results on PASCAL-$5^{i}$ between our method and other popular methods.

Method	$5^{0}$	$5^{1}$	$5^{2}$	$5^{3}$	avg
**Supervised approaches**					
CWT [34]	56.9	65.2	61.2	48.8	58.0
DAN [21]	54.7	68.6	57.8	51.6	58.2
MLC [40]	60.8	71.3	61.5	56.9	62.6
HSNet [7]	67.3	72.3	62.0	63.1	66.2
CyCTR [35]	69.3	72.7	56.5	58.6	64.3
IPMT [41]	71.6	73.5	58.0	61.2	66.1
MIANet [42]	68.5	75.8	67.5	63.2	68.7
**Self-supervised approaches**					
Saliency * [10]	51.5	49.1	48.1	39.0	46.9
MaskContrast * [10]	53.6	50.7	50.7	39.9	48.7
IPMT * [41]	57.9	57.2	55.4	43.9	53.6
MIANet * [42]	57.2	56.8	55.9	45.2	53.8
MaskSplit [11]	54.1	57.1	54.8	**46.1**	53.0
**Ours**	**58.4**	**57.9**	**58.7**	46.0	**55.1**

* represents the results obtained by adapting the methods used to assess the same settings.

**Table 2:** Comparison of results on COCO-$20^{i}$ and FSS-1000 between our method and other popular methods.

Method	$20^{0}$	$20^{1}$	$20^{2}$	$20^{3}$	avg	FSS-1000
**Supervised approaches**						
CWT [34]	30.3	36.6	30.5	32.2	32.4	
DAN [21]	-	-	-	-	24.4	85.2
MLC [40]	50.2	37.8	27.1	30.4	36.4	
HSNet [7]	37.2	44.1	42.4	41.3	41.2	86.5
PEFNet [43]	36.8	41.8	38.7	36.7	38.5	
MIANet [42]	42.5	53.0	47.8	47.4	47.7	
**Self-supervised approaches**						
Saliency * [10]	22.7	24.3	20.4	22.2	22.4	
HSNet [7]	29.3	25.6	20.5	23.0	24.6	76.1
MIANet * [42]	26.7	**27.2**	20.9	21.9	24.2	75.0
MaskSplit [11]	22.3	26.1	20.6	**24.3**	23.3	72.1 *
**Ours**	**37.4**	26.2	**21.3**	22.3	**26.8**	**78.1**

* represents the results obtained by adapting the methods used to assess the same settings.

**Table 3:** Comparative results on ISIC2018.

Method	mIoU
PATNet [45]	41.16%
PMNet [46]	51.2%
**Ours**	**65.2%**

**Table 4:** Computational complexity.

Method	FLOPs	Params	Number of Iterations	Time in Each Iteration
HSNet [7]	103.8 G	86.7 M	90	15 m
MLDAC (Ours)	112.0 G	96.1 M	18	15 m

**Table 5:** Ablation study on different layers of DACB.

$\frac{1}{8}$	$\frac{1}{16}$	$\frac{1}{32}$	Results
		✓	49.2
	✓	✓	53.5
✓	✓	✓	**55.1**

**Table 6:** Ablation experiments on the effectiveness of the proposed method on PASCAL-$5^{i}$.

Fixed Learnable PE	$\frac{1}{4}$ Connection	$\frac{1}{8}$ Connection	$\frac{1}{4}$+$\frac{1}{8}$ Connection	Results
		✓	✓	54.6
	✓	✓		54.3
✓	✓			54.1
✓		✓		53.9
✓			✓	54.5
✓		✓	✓	**55.1**
✓	✓	✓		54.8

## Data Availability

The data presented in this study are available upon request from the corresponding author. The data are not publicly available due to privacy concerns.
